# The Townshend Case

**Published:** 1906-08-25

**Authors:** 


### The Hospital

A Journal of

The Medical Sciences and Hospital Administration.

Vol. XL.—No. 1,040. SATURDAY, AUGUST 25, 1906.

### THE TOWNSHEND CASE.

The recent inquiry into the fitness of the Marquess Townshend to manage his affairs has presented a large number of curious phenomena both to the student of society and to the alienist. Upon those chiefly interesting to inquirers of the former class it is not our intention to dwell. The clerical "friend," and the legal father-in-law, and the "lady of title" who conspired with a civil servant to find a rich wife for a "consideration," all these people are by no means unworthy of notice by philosophers, but they may for the present be dismissed from our thoughts as mere examples of the principle that "wheresoever the carcase is, there will the eagles be gathered together." It is a matter of more importance to consider the result of the investigation by the light of the fact that there is no such thing as an isolated case, and that therefore, if it be true that the Marquess, although not of sufficient capacity to be trusted with the care of his property, is nevertheless fit to be trusted with the care of himself, there must be many other persons who are in similar condition, but who, nevertheless, are not subjected to similar restraints. We believe that no similar judicial inquiry has been held for many years, not since the once much-talked-of "Wyndham case"; and, if this be so, the fact is probably due to the enormous cost of the proceedings, as well as, partly, to the extremely unsatisfactory character of the possible result. The Marquess, as we understand the matter, is placed by the verdict, at the age of forty, very much in the legal position of a minor. If he cannot alienate his property, it is obvious that he would be unable to pledge his credit, unless, it might be, to such an extent as a jury might consider justifiable for "necessaries." It appears to be very doubtful whether he could be held answerable for any fraud or false pretence in relation to obtaining goods on credit. But it seems clear that he is entitled to exercise the functions of a legislator, and to record his vote in the House of Lords. It also seems clear that, if he were to become a widower, he would be entitled to marry again, and that, if his wife had children, his title and estates would descend to them. If he were indicted for a criminal offence, the question of his responsibility would be considered by a jury or by his peers. He seems to us to be left in a position at once anomalous and unenviable; but, nevertheless, in one which, if it be rightly accorded to him, should also be accorded to a large number of his fellow countrymen. There are hundreds of people who, in the homely language of some parts of England, are described as being "half-baked," but who, presumably because they are not peers of the realm, and neither very rich nor mad enough to be confined in asylums, are compelled to bear all the responsibilities which fall upon the capable, and are permitted, if such be their desire or tendency, to ruin themselves and their families in such fashion as may seem good to them.

The existence and conduct of persons of this class is of special interest to the community in two different ways; first, with regard to the disposal and employment of property in the limited circles immediately concerned, and, secondly, with regard to the probable character of any offspring which may proceed from the persons in question, and which, it is only too certain, are likely to deviate still more widely than their parents from a healthy standard of mind and body. It is not irrelevant to observe that the recent inquiry is not the first in which the holder of the Townshend peerage has been concerned. The third Marquess was a person of doubtful, and probably of female sex, whose so-called wife left him, and, without any divorce or legal annulment of her marriage, went through a second ceremony at Gretna Green with Mr. Margetts. Her eldest son assumed the name of Townshend and the courtesy title of Earl of Leicester, until he and the other descendants of the Gretna Green marriage were declared illegitimate by an Act of Parliament in 1842. The present Marquess is not a descendant of the third, but they spring, of course, from a common ancestor, and it is more than probable that they might both be accepted as interesting examples of the influence of heredity. Such examples among the offspring of the "half-baked" in a lower grade of society are familiar to parish doctors and relieving officers, as well as, not uncommonly, to magistrates and to the police. The existing laws relating to the insane are ripe for amendment in many important particulars; and, if the existence of persons who, like the Marquess Townshend, are only partially responsible for their actions, is to be recognised by the State, the time has certainly come at which their rights and disabilities should be laid down with the greatest attainable precision, and should be ascertainable by some simple and inexpensive process. In the case of persons of property, we might probably do worse than introduce into this country the French institution of the *Conseil de famille*, surrounded by such precautions as the differences between the two nations might require, but with authority to prevent either a legal marriage or the alienation of the family estate. With the poor the problem is even more complicated, because it is at once more important to the community, and far less easy, to prevent the indefinite multiplication of inferior types by irregular alliances. We once knew of an instance, in a provincial workhouse, in which a semi-imbecile woman, able to maintain herself in fine weather by fruit picking or light field work, always took her discharge early in the summer, and always returned pregnant early in the autumn. Her first labour was tedious, and the workhouse doctor had recourse to craniotomy, with the result that the necessity for this expedient, in her case, became a tradition of the institution, and was acted upon accordingly. In her eighth confinement, the arrival of the accoucheur was in some manner delayed, and the patient gave birth to a full-grown living child without any particular difficulty. It grew up an idiot; and, so far, may be held in some degree to have justified the practice pursued in relation to its seven predecessors.

